# A rare case of Sweet syndrome secondary to melioidosis

**DOI:** 10.1186/s12895-019-0096-2

**Published:** 2019-12-02

**Authors:** Sahathevan Vithoosan, Balendran Thanushah, Paramarajan Piranavan, Dayal Gamlaksha, Harindra Karunatilake, Ananda Jayanaga

**Affiliations:** 1 National Hospital of Sri Lanka, Colombo, Sri Lanka; 2 Internal Medicine, Saint Vincent Hospital, Worcester, MA 01608 UK

**Keywords:** Sweet syndrome, Melioidosis, Burkholderia pseudomallei

## Abstract

**Background:**

Melioidosis is an emerging infection in South Asia caused by *Burkholderia pseudomallei* with various clinical presentations that include pneumonia, bacteraemia, arthritis, and deep-seated abscesses. Various cutaneous manifestations have been described in association with melioidosis. However Sweet Syndrome secondary to melioidosis has not been reported in the literature. Herein we describe the first case of Sweet syndrome secondary to melioidosis.

**Case presentation:**

A 53-year-old previously healthy Sri Lankan female presented with high-grade fever, painful oral ulcers, odynophagia and multiple bilateral cervical lymphadenopathies for 1 month. She also had a loss of appetite and weight. She had oral ulcers and bilateral blepharitis. Dermatological examination revealed multiple tender papules with a mamillated appearance and targetoid lesions with a yellowish centre over the face, upper trunk and upper limbs. She also had multiple tender subcutaneous nodules over the extensor aspect of upper limbs. Her inflammatory markers were significantly elevated. Aspirate from a submental lymph node abscess revealed the growth of *Burkholderia pseudomallei.* Melioidosis antibody titer was > 10,240. The histology of the skin lesions of the face and left forearm showed a prominent neutrophilic infiltrate in the dermis and the morphological features were in favour of Sweet syndrome with panniculitis. She was started on intravenous meropenem 2 g daily and showed rapid clinical improvement with the disappearance of skin lesions as well as a reduction in inflammatory markers.

**Conclusion:**

Sweet syndrome is an uncommon inflammatory disorder known to be associated with upper respiratory tract and gastrointestinal infections, malignancies and the use of certain drugs. Melioidosis is an emerging infection with various cutaneous manifestations. This is the first case of melioidosis causing the secondary sweet syndrome. It emphasizes the importance of considering melioidosis as a potential aetiology in patients with Sweet syndrome.

## Background

Melioidosis is an emerging infection in South East Asia and Australia [1]. An increasing number of cases of melioidosis were reported in Sri Lanka in the recent past [2]. It has a wide range of clinical presentations, especially in the immunocompromised [3]. Clinical manifestations of melioidosis can be localized skin involvement or can be complicated with bloodstream invasion leading to sepsis and deep-seated abscesses [1]. Sweet’s syndrome also known as acute febrile neutrophilic dermatosis was first described by Robert Sweet in 1965. It can be either primary or secondary to various infections, haematological neoplasms, autoimmune diseases and drugs [4]. Varied cutaneous manifestations have been described in association with melioidosis [5]. However, Sweet syndrome secondary to melioidosis has not been described so far. Herein we describe a case of melioidosis associated with Sweet syndrome in a Sri Lankan female.

## Case presentation

**Fig. 1:**
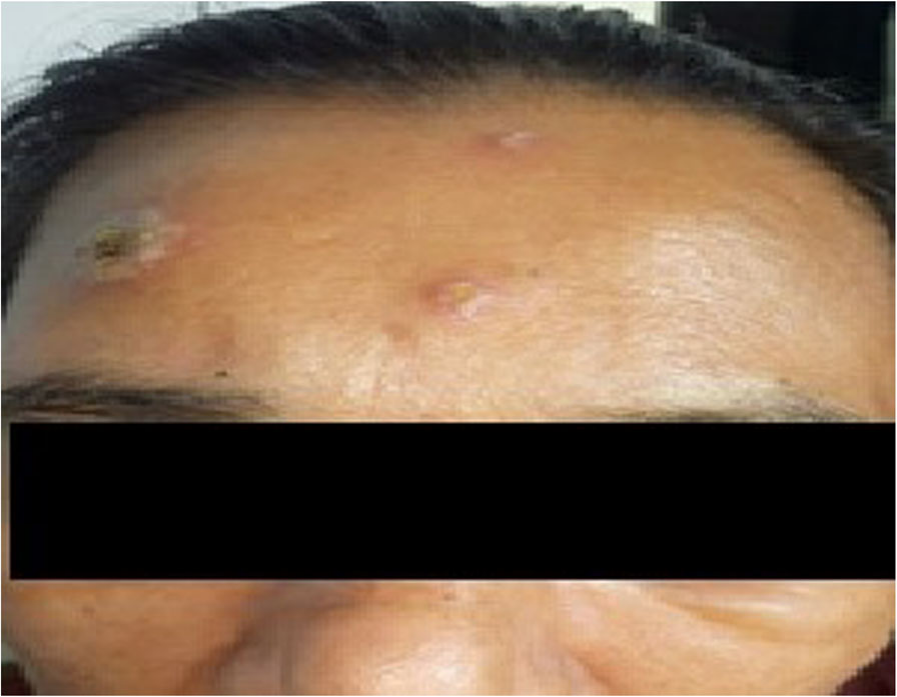
Targetoid skin lesions over the face

**Fig. 2:**
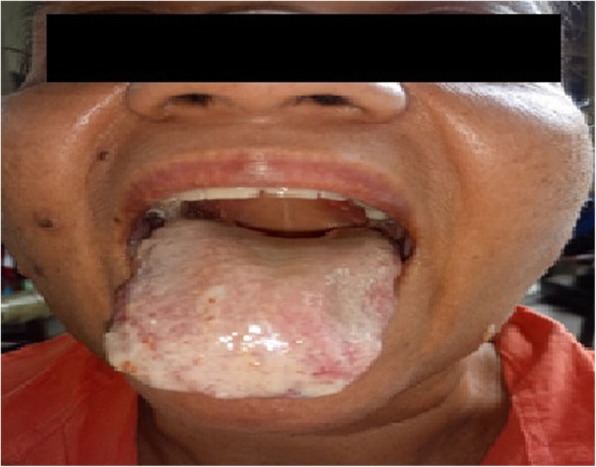
Mucosal lesions in the tongue

A 53-year-old previously healthy Sri Lankan female presented with high-grade fever, painful oral ulcers, odynophagia and multiple bilateral enlarged cervical lymph nodes for 1 month. She also had a loss of appetite and loss of weight. There was no cough or haemoptysis, past or contact history of tuberculosis, arthralgia, urinary symptoms or altered bowel habits. She denied high-risk sexual behaviour. On examination, apart from bilateral cervical lymphadenopathy she also had a discharging sinus over the submental region and bilateral blepharitis. Dermatological examination revealed multiple erythematous tender papules and plaques with the mamillated appearance and centrally yellowish targetoid lesions symmetrically distributed over the face (Fig. 1), upper trunk and upper limbs. The oral ulcers were multiple tender erosions with an erythematous border and a fibrinous base involving lips, tongue (Fig. 2) and buccal mucosa. She never experienced red eyes. She also had multiple tender subcutaneous nodules over the upper limbs. There was no hepatosplenomegaly. Cardiovascular, respiratory and neurological examinations, including the optic fundus, were normal.

The whole blood count showed mild neutrophil leucocytosis. ESR was 129 mm/1st hour (normal 0–20 mm/1st hour) and CRP was 195 mg/L (normal < 3 mg/L). Liver and renal function tests were normal. She had a positive mantoux test of 19 mm. The chest radiograph was normal. Repeated sputum examinations for Acid Fast Bacilli, Tuberculosis culture and GeneXpert were negative. Human immunodeficiency viral antigen and antibody were not detected. Her fasting blood sugar and HbA1C were normal. Blood culture remained sterile. Aspirate from the suppurative submental lymph node grew *Burkholderia pseudomallei (B. pseudomallei).* Melioidosis antibody titer was > 10,240.

**Fig. 3:**
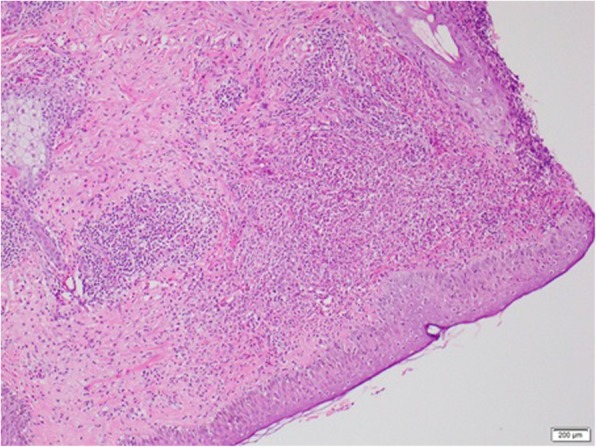
LM- H&E × 100-Diffuse and perivascular infiltrate of neutrophils in the dermis. Epidermis histologically unremarkable

**Fig. 4:**
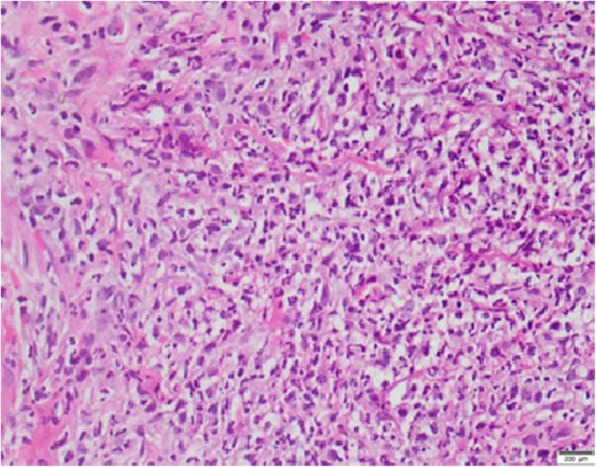
LM- H&E × 400-Diffuse neutrophil infiltrate with karyorrhectic debris in the dermis

Histology from skin lesions of the face and left forearm showed a prominent neutrophilic infiltrate in the dermis extending into the subcutaneous fat, which is in favour of Sweet syndrome with panniculitis (Figs. 3 and 4). Contrast-Enhanced Computerised Tomography (CECT) of the brain, chest, and abdomen revealed matted cervical and mediastinal lymph nodes, non-enhancing hypoechoic lesions in the liver and spleen and a right-sided pleural effusion with underlying consolidation.

The patient was started on intravenous meropenem 2 g daily. She showed rapid clinical improvement with the disappearance of skin lesions with a significant reduction in inflammatory markers. This case clearly demonstrates that, when sweet syndrome occurs in the background of systemic infection, it subsides with treating the infection alone with intravenous antibiotics. Our patient showed clinical resolution without requirements of steroids or other immunosuppressant medications.

## Discussion and conclusion

Melioidosis is caused by *B.pseudomallei* which is a free-living saprophyte in the environment and infects humans by direct penetration via skin abrasions or inhalation of dust [5]. It is a life-threatening infection that is estimated to cause 89,000 deaths per year worldwide [6]. The infection may remain localized or may progress rapidly through the bloodstream. The specific manifestations, severity, and chronicity of melioidosis may vary depending on bacterial strain and load, the route of bacterial entry (cutaneous penetration, ingestion or inhalation) and host immune function [6]. The various clinical manifestations of melioidosis are summarised in Table 1. Disseminated infections with multiorgan involvement complicating with sepsis, septic shock and death are more commonly seen in immunocompromised patients. The risk factors include but not limited to diabetes mellitus, organ failure (end-stage renal disease, cirrhosis, etc.), thalassemia, alcoholism, and immunosuppressive therapy [2]. However, like our patient melioidosis could also affect healthy adults and children without any obvious risk factors.

**Table 1:** Clinical manifestations and systems affected in melioidosis

System affected in melioidosis	Clinical manifestations
Skin and soft tissue	cellulitis, superficial and deep abscesses, ulcer, pustule
Musculoskeletal	septic arthritis, osteomyelitis, and muscle abscesses
Cardiovascular	endocarditis, Pericardial effusion
Respiratory	pneumonia, lung abscess
Abdominal	chiefly abscesses of the liver, spleen or psoas muscle
Central and peripheral nervous system	meningitis, subdural empyema, cerebral abscess, brain-stem encephalitis, transverse myelitis, Guillain Barré syndrome and status epilepticus
Genitourinary	urinary tract infection, prostatitis, abscess
Lymph nodes and salivary gland	abscesses
Other	Multiorgan involvement, bacteremia

Cutaneous melioidosis can present as an ulcer, pustule, cellulitis or a crusted erythematous lesion [5]. However, Sweet syndrome secondary to melioidosis has not been reported in the literature. Sweet syndrome is a rare dermatological condition characterized by an abrupt onset of fever, an elevated neutrophil count, tender erythematous skin lesions, and a diffuse infiltrate of mature neutrophils in the reticular dermis with oedema in the papillary dermis [7]. Upper respiratory tract and gastrointestinal infections are known to be associated with Sweet syndrome.

The possibility of the Sweet syndrome was suspected by the characteristic appearance of the skin lesions. Furthermore, the histology from the skin lesions revealed prominent collections of neutrophils and neutrophilic debris and lymphocytes in the dermis and subcutaneous fat without evidence of vasculitis helped to confirm our diagnosis. According to the modified classification criteria proposed by von den Driesch, diagnosis of Sweet’s syndrome requires the presence of both major and two out of four minor criteria [8]. Our patient met both major criteria and three minor criteria. Interestingly, even though oral mucosal involvement in classic Sweet’s syndrome is rare, our patient had extensive pseudomembranous like oral mucosal involvement.

Evaluating and working up for secondary sweet syndrome to rule out various potential etiologies such as malignancies, infections and autoimmune diseases was important as our patient did not have any recent exposure to new drugs. As a part of our initial workup, a lymph node biopsy was pursued in our patient. The lymph node biopsy histological examination excluded malignancies and cultures surprisingly revealed colonies of *B.pseudomallei.* A definitive diagnosis of melioidosis was made by culture of the causative organism from the lymph node aspirate and the high titer of melioidosis antibodies.

Sweet’s syndrome shows excellent response to treatment with systemic corticosteroids. Colchicine, dapsone and potassium iodide are the other first-line treatment options [9]. When Sweet syndrome is secondary to drugs the offending drugs should be discontinued. The underlying infection should be treated when Sweet syndrome is secondary to an infection. In the presence of an infection, treatment with steroids or other immune-suppressive drugs alone could be potentially harmful. She was treated with intravenous antibiotics and the lesions subsided with this alone without additional use of systemic steroids.

The case highlights the importance of considering melioidosis as a potential aetiology in patients with Sweet syndrome. Failure in clinical recognition and delayed laboratory diagnosis of melioidosis are not uncommon and could lead to treatment delays and poor outcomes. Mortality from melioidosis can exceed close to 40% in such cases [6]. Since both are rare conditions it is important for the clinician to be aware of this rare association. Unlike the idiopathic Sweet syndrome, here there is a remarkable improvement of the dermatological manifestations with the treatment of underlying melioidosis infection. It is important to look for the underlying cause for Sweet syndrome since the management can vary depending on the underlying aetiology.

## Data Availability

The datasets supporting the conclusions of this article are included in the article.

## Abbreviations

CECT: Contrast-Enhanced Computerised Tomography
CRP: C Reactive Protein
ESR: Erythrocyte Sedimentation Rate
